# Molecular insights into the effects of laser-induced optical breakdown (LIOB) after 1064 nm picosecond laser irradiation using a novel melanocyte-containing 3D skin model

**DOI:** 10.1007/s10103-025-04474-z

**Published:** 2025-05-13

**Authors:** Sebastian Huth, Yvonne Marquardt, Laura Huth, Peter Arne Gerber, Jens Malte Baron

**Affiliations:** 1 Department of Dermatology and Allergology, Uniklinik RWTH Aachen, Aachen, Germany; 2 Dermatologie am Luegplatz and Medical Faculty, Heinrich Heine University Düsseldorf, Düsseldorf, Germany; 3 Interdisciplinary Center for Laser Medicine, Uniklinik RWTH Aachen, Aachen, Germany

**Keywords:** 3D skin model, Wound healing, LIOB, Picosecond laser, Dexpanthenol

## Abstract

Picosecond lasers use a mechanism known as laser-induced optical breakdown (LIOB). However, the underlying molecular mechanisms are not yet fully understood. The aim of this study was to gain insights into the molecular effects of LIOB using novel melanocyte-containing 3D skin models. Since the threshold of LIOB depends on the melanin content of the skin, we established a new human 3D skin model comprising melanocytes. Irradiation was done with a diffractive optical elements (DOE-) assisted fractional 1064 nm Nd: YAG picosecond laser utilizing the energy setting of 0.2 J/cm^2^, with a spot size of 7 × 7 mm and one pulse per area. In a further approach, we post-treated the models topically with a dexpanthenol-containing ointment. Examination was done histologically and using next-generation sequencing. The histological analysis revealed intra-epidermal vacuoles with an intact environment immediately after irradiation of the models and even after 24 h. Post-treatment with the dexpanthenol-containing ointment accelerated the repair processes in the models, with vacuoles no longer visible after 24 h. We found an upregulation of matrix metalloproteinases, collagens, heat shock proteins, cytokines and chemokines, reflecting repair mechanisms and tissue remodeling after picosecond laser irradiation. Initial stimulation effects of laser therapy were maintained even after topical dexpanthenol post-treatment. We present the first in vitro study investigating the effects of LIOB after 1064 nm picosecond laser irradiation using a novel standardized melanocyte-containing 3D skin model. LIOB-induced intraepidermal vacuoles promoted skin regeneration processes, which could be supported and accelerated by post-treatment with a dexpanthenol-containing ointment.

## Introduction

The use of a fractional picosecond laser is an effective method for the treatment of a growing number of dermatological indications such as acne scars, striae, melasma, photo-damaged skin as well as for tattoo removal [1]. Picosecond lasers, including the neodymium: yttrium-aluminum-garnet (Nd: YAG) laser, are developed with a very short pulse duration and a high peak power density [2]. To reduce unwanted thermal damage, picosecond lasers are clinically used with micro-lens arrays (MLA) or diffractive optical elements (DOE), which allow the energy to be concentrated within laser microbeams [2, 3]. Following picosecond laser treatment of in vivo skin tissue, previous studies have shown the appearance of vacuoles in the epidermis resulting from a mechanism known as laser-induced optical breakdown (LIOB) [4–7]. During the laser pulse LIOB is triggered by the emission of accelerated seed electrons from laser-heated chromophores like melanin, which collide with surrounding molecules and thus release more free electrons [4]. As the density and energy of the free electrons increase, an ionized plasma forms which absorbs the remaining laser radiation from the pulse [4]. This hot plasma heats the surrounding tissue and creates a steam bubble that forms the intra-epidermal vacuoles [6]. In these vacuoles, only the small target pigments are removed without thermally damaging the adjacent tissue [8]. Previous studies revealed that LIOB formation depends on the irradiance of the laser and the color of the skin [8]. The higher the laser fluence and melanin concentration, the earlier LIOB formation begins [4, 7].

Although the physical process of LIOB formation is known, the molecular mechanism of action of the picosecond laser and the role the vacuoles play on dermal remodeling and neocollagenesis are still poorly understood [7, 9]. Previously published studies proved that 3D skin models are a reliable and standardized tool to study the molecular effects of various laser systems [10–16]. Since the presence of the skin chromophore melanin influences the threshold value of LIOB [8], we developed a novel standardized human full-thickness 3D skin model containing melanocytes.

The aim of this study was to gain insights into the molecular effects of the dermal regeneration after picosecond laser treatment using novel standardized melanocyte-containing 3D skin models.

## Materials and methods

### Isolation and cell culture of normal human epidermal keratinocytes (NHEK) and normal human dermal fibroblasts (NHDF)

Primary NHDF and NHEK cells were isolated and cultivated as described previously [16–18].

### 3D skin equivalents containing melanocytes

The dermal part of the skin equivalents was constructed as previously described [16] by merging bovine collagen I solution (Vitrogen, Cohesion Technologies, Palo Alto, CA, USA) and 10x concentrated Hank’s balanced salt solution (Gibco/Invitrogen, Darmstadt, Germany) (ratio 8:1). After neutralization with 1 M NaOH, 1 × 10^5^ NHDF cells were added and poured into polycarbonate cell culture inserts (3 μm pore size, Nunc; Thermo Fisher, Waltham, USA). After two days of incubation at 37 °C and 5% CO_2_, 2 × 10^6^ NHEK cells and 0.5 × 10^6^ normal human primary epidermal melanocytes (Lifeline Cell Technology, Frederick, USA) were seeded on the dermal equivalent. On the following day, skin equivalents were lifted to the air-liquid interface (ALI).

### Laser treatment and study design

Laser treatment of skin models was performed with a DOE-assisted 1064 nm Nd: YAG picosecond laser utilizing the energy setting of 0.2 J/cm^2^, with a spot size of 7 × 7 mm and one pulse per area (PicoStar picosecond laser with MicroSpot handpiece, Asclepion Laser Technologies GmbH, Jena, Germany). The pulse duration is 300 picoseconds and it is not adjustable. The settings applied were taken from the manufacturer’s manual for the treatment of human skin with the MicroSpot handpiece. The value 0.2 J/cm² is well tolerated by different skin types.

In a further approach, we post-treated the models topically with a dexpanthenol-containing wound care ointment (Bepanthen wound and healing ointment; Bayer Vital, Leverkusen, Germany). Experiments were performed three times independently in triplicates.

### Light microscopy

For light microscopy, 5 μm cryosections of 3D skin models were embedded in Tissue-Tek O.C.T. ™ compound (Sakura Finetek), stained with haematoxylin and eosin (H&E) and subsequently examined by a photomicroscope (DMIL, Leitz, Wetzlar, Germany).

### Immunofluorescence analysis

For immunofluorescence, 5 μm cryosections were fixed for 10 min in acetone at 4ºC. First antibody MMP-9 (ab76003, Abcam, Cambridge, UK), was diluted with Antibody Diluent (Dako, Glostrup, Denmark) and incubated at room temperature for 1 h. Following the washing steps with PBS, the sections were incubated in fluorochrome-conjugated secondary antibody Alexa Fluor 488 IgG H + L (Molecular Probes, Eugene, Oregon, USA) for 1 h at room temperature. Cell nuclei were stained with DAPI (Applichem, Darmstadt, Germany). After a final washing step, sections were mounted in Fluorescent Mounting Medium (Dako) and coverslipped. The sections were stored in the dark at 4ºC. Microscopy was performed with photomicroscope (DMIL) equipped with epifluorescence illumination and digitally photodocumentation (DISKUS, Hilgers, Königswinter, Germany). Densitometric measurements of staining intensities in clearly defined reference areas (quantitative immunohistomorphometry) was performed using the ImageJ software (National Institute of Health, Bethesda, USA).

### Fontana-Masson staining

Melanin content was determined using the Fontana-Masson staining kit (HT200; Sigma-Aldrich, St. Louis, USA), according to the to the manufacturer’s instructions.

### Next generation sequencing

Total RNA from 3D skin models was extracted with the Nucleo Spin RNA Kit (Macherey and Nagel, Düren, Germany), according to the manufacturer’s instructions. RNA quality control was done for all samples using a Bioanalyzer (Agilent, Santa Clara, USA). Lexogen QuantSeq 3’mRNA-Seq v2 Library Prep Kit FWD with UDIs (Lexogen GmbH, Vienna, Austria). A NextSeq 500/550 Mid Output Kit v2.5 (150 cycles) was used. ERCC RNA Spike-in Mix (Thermo Fisher) was employed. The generation of the FASTQ files was performed by bcl2fastq (Illumina, San Diego, USA). To facilitate reproducible analysis, samples were processed using the publicly available nf-core/RNA-seq pipeline version 3.12.0 [19] implemented in Nextflow 23.04.1 [20] using Docker version 24.0.2, build cb74dfc with the minimal command. In brief, lane-level reads were trimmed using Trim Galore 0.6.7 and aligned to the human genome (GRCh38.p13) using STAR 2.7.9a [21]. Gene-level and transcript-level quantification was done by Salmon 1.10.1 [22]. Differential expression analysis was performed using custom scripts in R version R version 4.2.2 (2022-10-31) using the DESeq2 1.28.0 *framework* [23]. RNA seq data availability is provided in the data availability statement.

### Statistical analysis

Data are given as arithmetical means ± standard deviation (SD). Mann-Whitney U test was performed with GraphPad PRISM version 7 (La Jolla, CA, USA). Values of *p* < 0.05 were considered statistically significant.

## Results

**Fig. 1:**
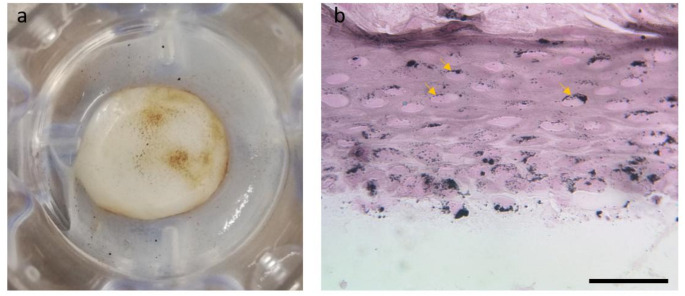
Novel melanocytes-containing 3D skin models exhibit a pigmentation. **(a**) Macroscopic examination of melanocyte-containing 3D skin models showing a brown pigmentation. **(b)** Fontana-Masson staining detected melanin in 3D skin models. Arrowheads indicate areas of pigment accumulation in keratinocytes. Representative images of three independent experiments performed in triplicates are shown. Magnification = 400x, scale bar = 50 μm

Macroscopic images of the newly developed melanocytes-containing 3D skin models showed at least partially a clear pigmentation (Fig. 1a) and Fontana-Masson staining revealed an accumulation of melanin in the keratinocytes within the entire epidermal equivalent (Fig. 1b).

**Fig. 2:**
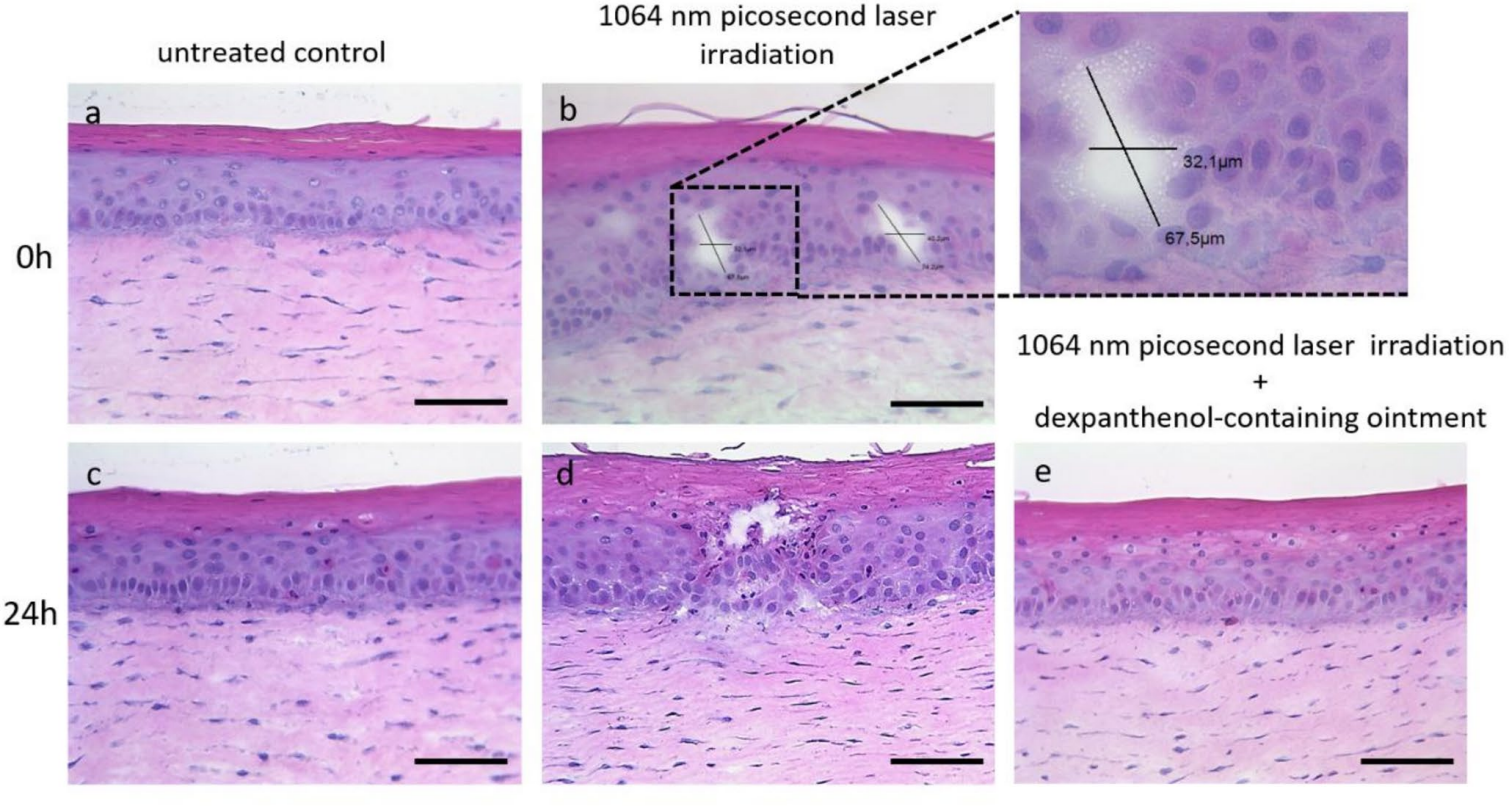
Diffractive optical elements (DOE)-assisted fractional 1064 nm Nd: YAG picosecond laser irradiation induced vacuole formation in 3D skin models. Representative hematoxylin and eosin stained cross-sections of 3D skin models. **(a)** Untreated controls for comparison with laser irradiated models. **(b)** 3D skin models treated with a 1064 nm Nd: YAG picosecond laser exhibited intra-epidermal vacuoles immediately after irradiation. Rectangle region is separately enlarged and shows the measured dimensions of the vacuole. **(c)** Untreated control models after 24 h. **(d)** 3D skin models treated with a 1064 nm Nd: YAG picosecond laser exhibited intra-epidermal vacuoles 24 h after irradiation. **(e)** 3D skin models treated with a 1064 nm Nd: YAG picosecond laser and post-treated with a dexpanthenol-containing ointment showed a fully restored epidermal equivalent after 24 h. Representative images of three independent experiments performed in triplicates are shown. Magnification = 200x, scale bar = 100 μm

Immediately after 1064 nm picosecond laser irradiation with DOE, histological analysis of H&E-stained cross-sections showed clearly defined intra-epidermal vacuoles in the 3D skin models compared to non-irradiated controls (Fig. 2a + b). No vacuoles were detected in models that did not contain melanocytes (data not shown). The location of these vacuoles varied from stratum granulosum to stratum basale without any damage to surrounding cells. After 24 h histology demonstrated a beginning wound closure of the vacuoles (Fig. 2d). In 3D skin models that received a post-treatment with dexpanthenol-containing ointment immediately after the laser treatment, no vacuoles were visible after 24 h (Fig. 2e).

**Fig. 3:**
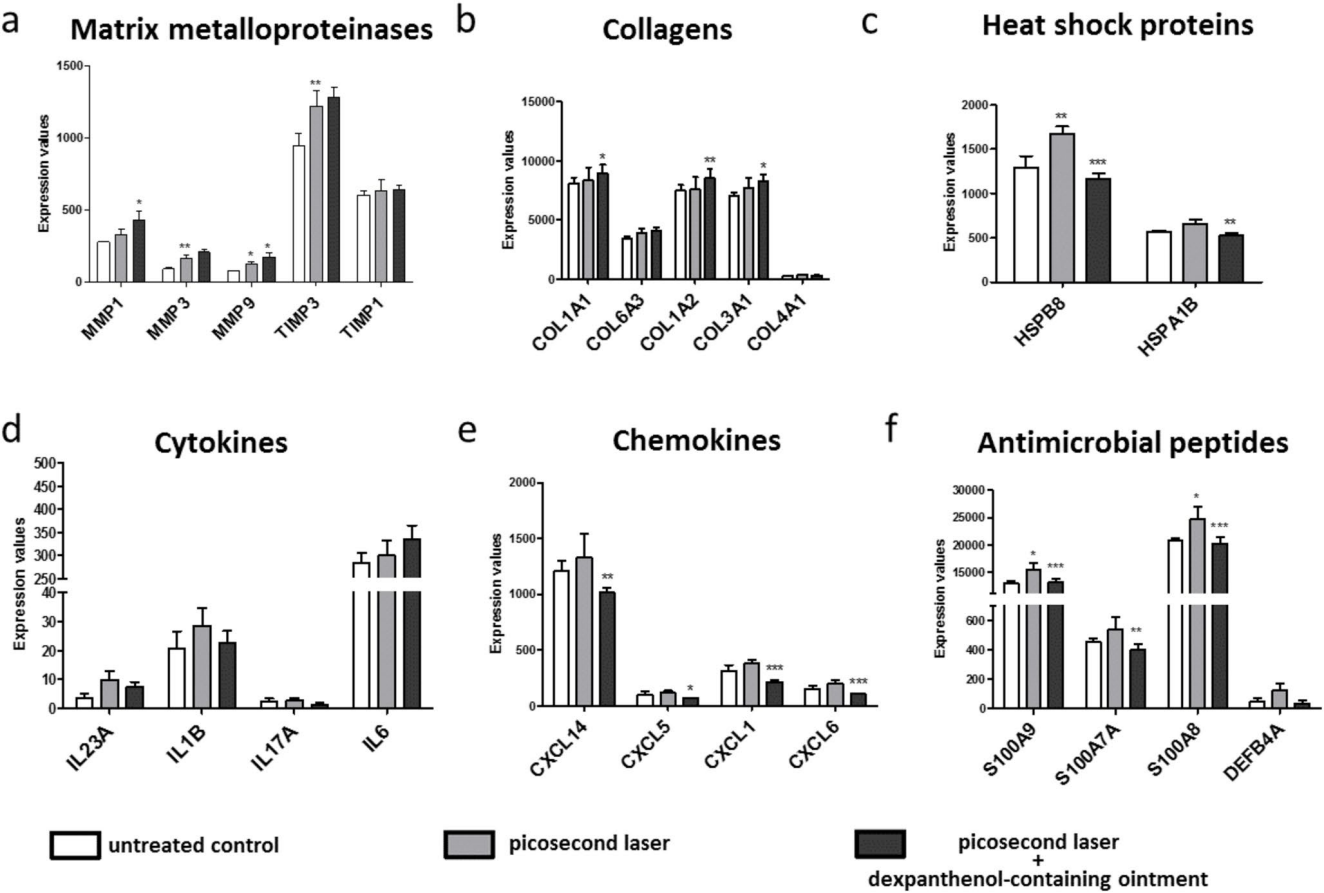
Gene expression data reflect the wound-healing promoting effects of the vacuole formation after LIOB induction by picosecond laser irradiation. Next-generation sequencing analysis detected upregulated mRNA levels of **(a)** matrix metalloproteinases, **(b)** collagens, **(c)** heat shock proteins, **(d)** cytokines, **(e)** chemokines and **(f)** antimicrobial peptides in 1064 nm Nd: YAG picosecond laser irradiated 3D skin models (grey columns) compared to untreated controls (white columns). Post-treatment with a dexpanthenol-containing ointment (black columns) increased the expression of matrix metalloproteinases, tissue inhibitor of metalloproteinases and IL6 and reduced the expression of heat shock proteins, cytokines, chemokines and antimicrobial peptides, compared to picosecond laser-irradiated 3D skin models without dexpanthenol-containing ointment post-treatment. Asterisks above the grey columns indicate a significant regulation in 1064 nm Nd: YAG picosecond laser irradiated 3D skin models compared to untreated controls and asterisks above the black columns indicate a significant regulation in 1064 nm Nd: YAG picosecond laser irradiated 3D skin models that were post-treated with the dexpanthenol-containing ointment compared to only picosecond laser irradiated models. Three samples were analyzed per condition. Data are given as arithmetical means ± standard deviation; *p < 0.05, **p < 0.01, ***p < 0.001

Next, we performed next generation sequencing to investigate the molecular effects of picosecond laser treatment. Bioinformatic analysis revealed an upregulation of matrix metalloproteinases (MMP1, MMP3, MMP9) and their inhibitors (TIMP3, TIMP1; Fig. 3a), collagens (COL1A1, COL6A3, COL1A2, COL3A1, COL4A1; Fig. 3b), heat shock proteins (HSPB8, HSPA1B; Fig. 3c), cytokines (IL23A, IL1B, IL17A, IL6; Fig. 3d), chemokines (CXCL14, CXCL5, CXCL1, CXCL6; Fig. 3e) as well as antimicrobial peptides (S100A9, S100A7A, S100A8, DEFB4A; Fig. 3f) after picosecond laser irradiation compared to non-irradiated controls. Post-treatment with a dexpanthenol-containing ointment led to an increased expression of matrix metalloproteinases, tissue inhibitor of metalloproteinases and IL6 on the one hand, and to a reduced expression of heat shock proteins, cytokines, chemokines and antimicrobial peptides on the other hand, compared to picosecond laser-irradiated 3D skin models without dexpanthenol-containing ointment post-treatment (Fig. 3a-f).

**Fig. 4:**
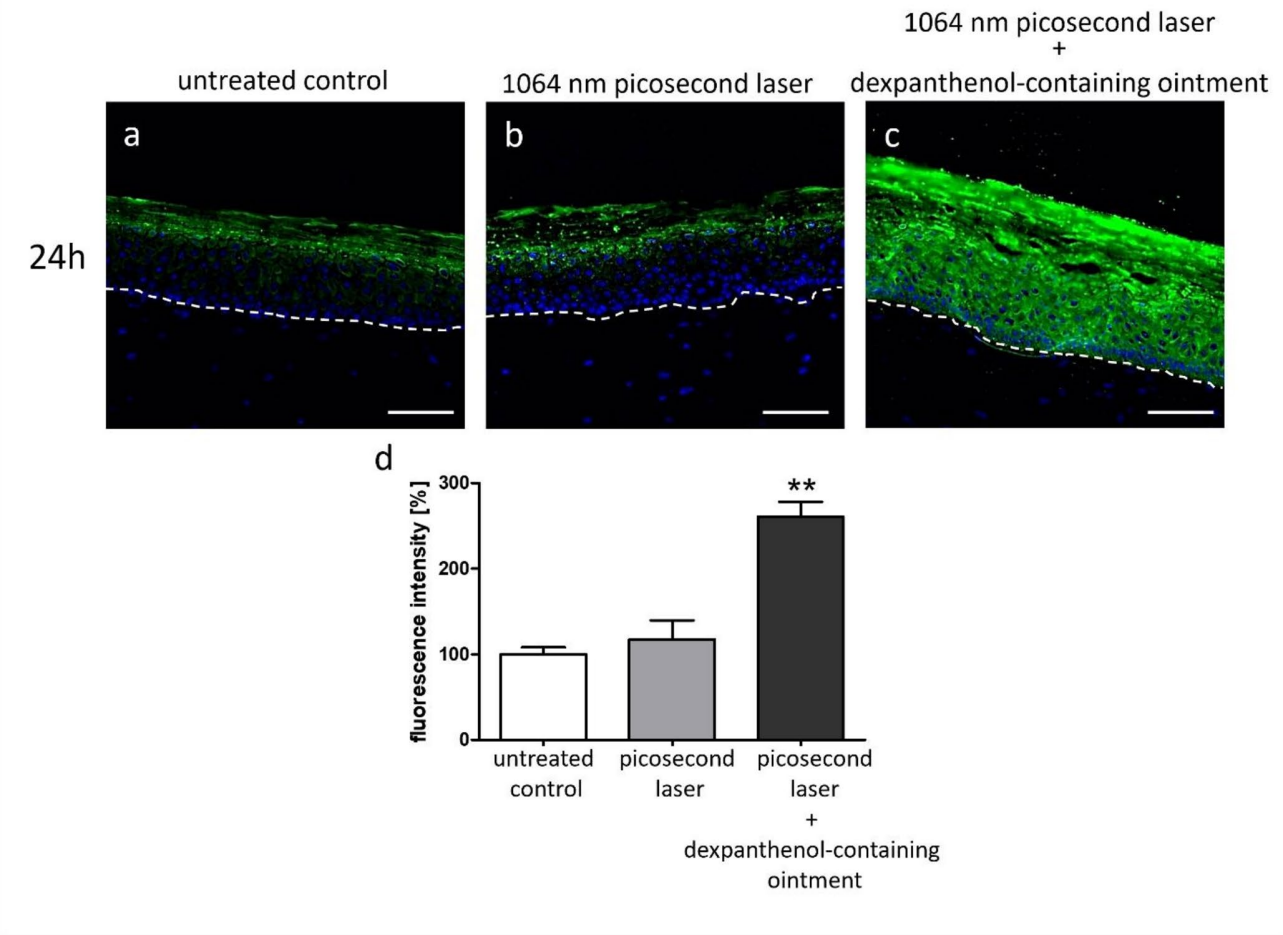
1064 nm Nd: YAG picosecond laser irradiation promotes MMP9 expression, which can be further enhanced by post-treatment with a dexpanthenol-containing ointment. Representative images of immunofluorescence staining with a MMP9 antibody of **(a)** an untreated control, **(b)** a 3D skin model irradiated with 1064 nm Nd: YAG picosecond laser and **(c)** a 3D skin model irradiated with 1064 nm Nd: YAG picosecond laser and post-treated with a dexpanthenol-containing ointment. Three independent experiments were performed in triplicates. Green fluorescence indicates MMP9 expression, while counterstaining was done with DAPI (blue). Dashed line shows the basal membrane. Magnification = 200x, scale bar = 100 μm. **(d)** Quantification of fluorescence intensity measured at three representative positions per image of all three experiments. Fluorescence intensity of the untreated control was defined as 100% and the other samples were normalized to it. Data are given as arithmetical means ± standard deviation; **p < 0.01

At the protein level, immunofluorescence staining showed a moderately increased expression of wound healing modulator MMP9 after picosecond laser treatment compared to non-irradiated controls (Fig. 4a + b). This effect was further enhanced by post-treatment with the dexpanthenol-containing ointment (Fig. 4c). Quantitative fluorescence measurement revealed a highly significant increase in MMP9 fluorescence intensity only in laser-irradiated 3D skin models that were additionally treated with the dexpanthenol-containing ointment compared to untreated controls and picosecond laser-irradiated 3D skin models without dexpanthenol-containing ointment post-treatment (Fig. 4d).

## Discussion

Picosecond lasers build a new group of laser devices characterized by an ultrashort pulse duration [24]. The tissue effects of such lasers are based on the chromophore-assisted formation of ionized plasma - a physical process known as LIOB [4]. Previous studies have shown the development of intra-epidermal vacuoles resulting from the disappearance of the plasma [4, 6, 7]. It has been speculated that this vacuole formation yields to dermal remodeling due to cell signaling and cytokine release [4, 7, 9, 24]. However, the detailed molecular changes that lead from the localized damage after LIOB to the remodeling processes are still poorly understood.

We present the first in vitro study using human full-thickness 3D skin models to investigate the molecular effects of the LIOB formation on skin regeneration. Such in vitro 3D skin models highly simulate the human skin in terms of tissue structure, gene expression and metabolic activities [25]. In this context, previous studies have shown that 3D skin models are a reliable and standardized tool to study the molecular effects of different laser systems [10–13, 15]. Since melanin is the predominant chromophore in the skin that contributes to the LIOB process [1], we developed new standardized 3D skin models including melanocytes and could prove that these in vitro models exhibit a light pigmentation where melanin has been deposited in the keratinocytes within the entire epidermal equivalent. This corresponds to the in vivo situation in which melanin is transferred from the melanocytes to the keratinocytes to exert its photoprotective function in these cells.

Our histological analyses revealed that the DOE-assisted fractional 1064 nm Nd: YAG picosecond laser irradiation produced intra-epidermal vacuoles immediately after treatment when melanin was deposited in our models. These vacuoles revealed a similar morphology and size to those vacuoles in human skin [6, 7, 26]. Former studies postulated that also hemoglobin can act as a chromophore to induce the LIOB process [27]. Since there are no blood vessels in the in vitro 3D skin model we used, our data support previous assumptions that a sufficient amount of melanin in the epidermis is enough to trigger the LIOB effects [6]. These data demonstrate that a melanocyte-containing 3D skin model can serve as a standardized in vitro tool to study the molecular effects of LIOB. After 24 h these microinjuries were still visible with a beginning closing of the wound-edge. A histologic appearance of vacuoles even after 24 h is consistent with in vivo studies [4].

Previous studies have identified an increased production of elastic fibers, skin collagen, and mucin in human skin and a stimulated expression of collagen markers and cytokines in murine skin after picosecond laser irradiation [9, 28]. Now performing a whole transcriptome analysis using next-generation sequencing, we provide comprehensive insights into the molecular effects of picosecond laser irradiation using in vitro 3D skin models.

After picosecond laser treatment, we found an upregulated expression of cytokines (e.g. IL1B), chemokines (e.g. CXCL5) and antimicrobial peptides (e.g. S100A9), which play prominent roles particularly in the early phase of wound healing [29, 30]. These results confirm previous hypotheses that chemokines and cytokines are responsible for regulating the injury response after LIOB induction [4].

In addition, we observed an increased expression of heat shock proteins (e.g. HSPB8), which are stress-inducible proteins that maintain cellular proteostasis and protect cells from stress [31]. These results are consistent with studies reporting an increased expression of HSPs in mouse skin following picosecond laser irradiation and suggest that the thermal damage caused by the laser stimulates collagen folding and secretion via HSP activity [9].

We also found a stimulated expression of collagens (e.g. COL1A1), which molecularly supports previous studies linking epidermal vacuoles to the formation of new collagen [7, 28]. These findings are also in line with animal studies detecting levels of collagen synthesis markers after picosecond laser treatment [9]. As recent studies have shown, the crosstalk between dermal fibroblasts and macrophages in the skin is of significant importance for the regulation of collagen expression in the fibroblasts [32]. It is therefore likely that the stimulatory effects of LIOB shown in this study are even stronger in the presence of macrophages. For technical reasons, however, simultaneous co-culture of keratinocytes, fibroblasts, melanocytes and macrophages in a 3D skin model is not yet possible.

Moreover, mRNA and protein levels of matrix metalloproteinases (e.g. MMP1 and MMP9), whose impact on matrix remodeling is well known [33], were increased in our models after picosecond laser treatment. While MMP1 expression is induced in injured skin when keratinocytes bind type I collagen at the wound-edge to facilitate keratinocyte migration [34], animal studies suggest that MMP9 plays an important role in scarless wound healing [35]. In total, these gene expression data reflect the wound-healing promoting effects of the vacuole formation after LIOB induction by picosecond laser irradiation.

In a further approach, we wanted to investigate whether post-treatment with a routinely applied topical treatment that promotes wound healing could be beneficial for picosecond laser treatment. In this context, previous in vitro and in vivo studies have shown the usefulness of topical dexpanthenol treatment in wound healing after laser therapy [15, 16, 36–38]. Histologically, post-treatment with a dexpanthenol-containing ointment reduced the downtime in our models by showing a fully restored epidermal equivalent with completely disappeared vacuoles after 24 h.

At the molecular level, post-treatment with a dexpanthenol-containing ointment led to an increased expression of matrix metalloproteinases, their inhibitors and IL6. These findings are in line with previous data showing that dexpanthenol exerts its wound healing promoting effects at least partly via MMP and IL6 stimulation [14, 16, 37]. Although described as a pro-inflammatory cytokine, previous studies emphasized a role of IL6 in wound healing by showing that IL6 knockout mice suffer from delayed cutaneous wound healing [39].

Furthermore, we observed a reduced expression of heat shock proteins, cytokines, chemokines and antimicrobial peptides after dexpanthenol treatment compared to picosecond laser-irradiated 3D skin models without topical dexpanthenol post-treatment. Interestingly, dexpanthenol appears to exert its well-known anti-inflammatory effects [36] here without impairing the skin-regenerating effect of LIOB. Further studies investigating these observations in depth are warranted.

One limitation of the human in vitro 3D skin model used in this study is that it certainly cannot fulfill all the requirements of in vivo conditions, such as vascularization. However, it represents a simplified model that enables comparable and reproducible data in vitro, while allowing histological and gene expression analyses at RNA and protein level at different time points, which is hardly possible in clinical in vivo studies in humans due to ethical reasons.

## Conclusion

This is the first in vitro study to investigate the molecular effects of LIOB after picosecond laser treatment using a newly developed standardized human melanocyte-containing 3D skin model. Our results support previous in vivo and ex vivo studies showing that LIOB is generated after picosecond laser treatment, leading to intra-epidermal vacuoles in the epidermal part of our models. This vacuole formation in the models is associated with a gene expression pattern that stimulates skin remodeling. Post-treatment with a dexpanthenol-containing ointment can positively support these molecular effects and reduce the downtime after laser treatment.

## Data Availability

The datasets generated during the current study are available from the corresponding author upon reasonable request.
